# Benefits of an Orthopedic Education Research Collaborative: An Innovative Approach

**DOI:** 10.7759/cureus.34903

**Published:** 2023-02-12

**Authors:** Eli B Levitt, Kyle D Paul, Sohrab K Vatsia, Brian Scannell, Joshua C Patt, Kim Templeton, Gerald McGwin, Brent A Ponce

**Affiliations:** 1 Orthopedic Surgery, Herbert Wertheim College of Medicine, Florida International University, Miami, USA; 2 Internal Medicine, Palmetto General Hospital, Hialeah, USA; 3 Orthopedic Surgery, Heersink School of Medicine, University of Alabama at Birmingham, Birmingham, USA; 4 Orthopedic Surgery, Joe R. and Teresa Lozano Long School of Medicine, University of Texas Health Science Center, San Antonio, USA; 5 Orthopedic Surgery, Hughston Clinic, Columbus, USA; 6 Orthopedic Surgery, Atrium Health, Charlotte, USA; 7 Orthopedic Surgery, University of Kansas School of Medicine, Kansas City, USA; 8 Epidemiology, Heersink School of Medicine, University of Alabama at Birmingham, Birmingham, USA

**Keywords:** collaborative, research, surgical education, residency, orthpaedics

## Abstract

Background

Graduate Medical Education (GME) research in orthopedic surgery is an important but underrepresented subject in the medical literature. It was unknown if orthopedic residency leaders were interested in a surgical education research collaborative (*orthopedic collaborative*). The objectives of this study were to assess the potential benefit of an orthopedic collaborative from orthopedic residency leaders and investigate the factors associated with the support of a research collaborative within a surgical subspecialty.

Methodology

An anonymous 19-question survey-based study was distributed through REDCap (Nashville, TN, USA) to orthopedic residency leaders in the United States, from July to October 2020. The main outcome was perceived benefit. Additional aspects included program characteristics, challenges in performing resident education research, and organizational issues such as authorship, frequency of study requests, and governance.

Results

Almost all orthopedic faculty leadership (99%, 73/74) stated that resident education and faculty development research projects would benefit from an orthopedic education research collaborative. In comparison to unsupportive respondents, younger age (*P* = 0.006), 15 or fewer years in practice (*P *= 0.04), and having 0 to 100 peer-reviewed publications (*P* = 0.047) were associated with support for an orthopedic collaborative.

Conclusions

Challenges related to survey-based study quality and generalizability at single institutions can benefit from multi-institutional collaboration to develop high-quality studies that capture a representative sample to support orthopedic surgery program development.

## Introduction

Graduate Medical Education (GME) research is an important but underrepresented subject in the literature of many surgical subspecialties [1,2]. The importance of scholarly activity and educational content in orthopedic training is supported by national groups, including the Accreditation Council of Graduate Medical Education [3], the American Orthopedic Association [4], and the American Academy of Orthopedic Surgeons [5]. In 2021, Hogan et al. [6] described the value of research during orthopedic training for programs, trainees, and faculty. At the time of this study, there were regional/national groups that focused on conducting orthopedic clinical trials and the Science of Variation Group directed survey-based studies on orthopedic practices [7-9]; however, there were no peer-reviewed publications reflecting the topic of collaboratives in education research within orthopedics.

While initiatives to promote GME research face challenges, including scarce funding, time constraints, and limited experience [10,11], communities of individuals devoted to GME research in other specialties have successfully overcome these issues to produce high-quality research [12,13]. The potential benefits of an orthopedic collaborative match the recommendations described by Hogan et al. [6]. Since 2011, models for collaboratives within other fields of medicine demonstrated the utility of collaboration [1,4,12,14-16]. Reported benefits of collaboratives include improved scholarly activity, improved physician wellness, and increased patient safety [17-20]. For example, the Best Evidence Medical Education (BEME) collaboration is an international group of individuals, universities, and professional organizations committed to the development of evidence-informed education in the medical and health professions [21]. Additionally, the Society for Improving Medical Professional Learning (SIMPL) collaborative has developed a mobile application at the forefront of surgical quality improvement - tailoring a data-driven platform for individualized operative performance feedback at over 70 member institutions [22]. BEME and SIMPL have both identified utility in faculty development programs to further resident education and have demonstrated potential for improvement in the field of GME research [22,23]. Improved study quality through collaborative efforts may also help reduce survey/respondent fatigue [24], the phenomenon of over-surveying that leads to diminishing response rates, and the other well-known challenges involved with survey studies, including appropriate length, meaningful purpose, and transparency [24,25]. Innovative research practices represent a unique way to overcome the challenges in research and support leadership development [14,17].

Although other surgical specialties have established education collaboratives, it was unknown if orthopedic residency leaders were interested in establishing an orthopedic education research collaborative (“orthopedic collaborative”). The primary objective of this study was to assess the potential benefit of an orthopedic collaborative, estimate the ability to perform multi-institutional, survey-based education research, and investigate the factors associated with support for a collaborative within orthopedics. The authors hypothesized that individuals with more academic/research experience would support orthopedic education research collaborative as a positive potential avenue to perform education research.

## Materials and methods

Study design, setting, and participants

An invitation to participate in an anonymous, 19-question, survey-based study was distributed to the orthopedic surgery residency program leadership of the 129 American Orthopedic Association/Council of Orthopedic Residency Directors (AOA/CORDs) in the United States. Survey questions were developed and formatted to minimize response bias. The survey was sent to orthopedic surgery residency Program Directors (PDs) at each institution, electronically, utilizing contact information obtained from the Accreditation Council for Graduate Medical Education (ACGME), and responses were collected between July and October 2020. An effort was made to maximize the response rate through three email reminders. To understand the need for an orthopedic collaborative, this study investigated the opinions of orthopedic residency leaders and the association between factors and support for an orthopedic education research collaborative. This study was approved by an Institutional Review Board with exempt determination.

Outcomes and measures

This study collected demographic information, education research experience, opinions regarding concerns and challenges related to performing education research, and the governance of a potential orthopedic education research collaborative. Characteristics included age, gender, state of the residency program, program size, years in practice, academic rank, and leadership position(s). Academic rank was specifically subcategorized according to the titles of Professor, Associate Professor, Assistant Professor, and Clinical Faculty. Of note, the title *Clinical Faculty* was utilized to capture members of orthopedic residency leadership who are unaffiliated with a university hospital or academic medical center. Leadership positions encompassed the roles of Chair, Vice Chair, PD, Other, and No Response. The role *Other* was utilized to record the position of Departmental Chief, Subspecialty Director, Quality Chief, and Research Director. Clinical Information regarding research experience included several peer-reviewed citations, participation in orthopedic subspecialty collaborative groups, challenges about orthopedic resident education, and concerns with the current state of orthopedic resident education. The last section of the survey sought to solicit recommendations and assess potential benefits for a potential orthopedic education research collaborative. The study was administered using REDCap (REDCap, Nashville, TN, USA) [26]. Survey questions are available in the supplemental digital content (Appendix).

Statistical analysis

The American Association for Public Opinion Research guidelines were followed for best practices for survey research evaluation [27]. Specifically, an electronic survey was chosen to optimize distribution and confidentiality, with questionnaire design and sample selection constructed based on minimizing response bias and maximizing generalizability to orthopedic surgery training programs across the United States. Descriptive statistics were compiled for the faculty and program characteristics. Tests of association among the groups were completed using chi-square or Fisher’s exact test for categorical data grouped by those who would support orthopedic education research collaborative versus those who were unsupportive. One-way analysis of variance was used to measure differences between the groups for continuous measurements. Additional chi-square analysis was utilized to compare the responses of men and women. Analyses were conducted using SAS Version 9.4 and JMP Version 14.0 (SAS Institute Inc., Cary, NC, USA).

## Results

Respondent characteristics and program information

A total of 74 orthopedic residency faculty (20% women; age, mean ± SD, 48 ± 9 years, range 35-75 years) completed the survey. PDs represented 84% (62/74) of the total responses from faculty, with an overall program response rate of 48% (62/129 potential programs). Additional faculty leadership roles included Department Chairs (3%, 2/74) and Vice Chairs (3%, 2/74). Four individuals were both the Chair and PD. Ten of the respondents were both the PD and Vice Chair. Responses represented a broad geography across the United States, with 23% Northeast, 34% South, 24% Midwest, and 19% West. Approximately two in three programs (68%, 50/74) had an average number of four to six residents per year. Small programs (two to three residents per year) represented 19% of the group, while larger programs (seven or more residents per year) represented 14% of the group. Respondents had been in practice (post-training) for a mean of 16.0 ± 9.0 years, and nearly two in three (64%, 47/74) reported 0 to 30 PubMed citations (i.e., peer-reviewed publications). One segment of the group (30%, 22/74) reported 31 to 100 peer-reviewed publications, and another segment of the group (7%, 5/74) reported between 101 and 400 peer-reviewed publications. Faculty characteristics and program information can be found in Table 1.

**Table 1:** Orthopedic faculty characteristics and program information. *Current leadership roles total is greater than 100% because individuals were able to select one or more survey choices. Other leadership roles included Chief of a department, Director of a program/subspecialty, Quality Chief, and Research Director.

Characteristic (*N* = 74)	No. (% of total)
Age (years), mean ± SD	48.2 ± 8.8
Age groups (years)	
35-44	31 (42)
45-54	26 (35)
55+	17 (23)
Gender	
Women	15 (20)
Men	59 (80)
Region	
Northeast	17 (23)
South	25 (34)
Midwest	18 (24)
West	14 (19)
Program size (average number of residents per year)	
2-3	14 (19)
4-6	50 (68)
7 or more	10 (13)
Years in practice (post-training), mean ± SD	16.0 ± 9.0
Academic rank	
Clinical Faculty	4 (5)
Assistant Professor	16 (22)
Associate Professor	37 (50)
Professor	17 (23)
Current Leadership Roles*	
Program Director	62 (84)
Chair, only	2 (3)
Vice Chair, only	2 (3)
Other leadership roles, only	3 (4)
No response	5 (7)
Chair and Program Director*	4 (5)
Vice Chair and Program Director*	10 (14)
Other leadership roles*	8 (11)
Total number of PubMed citations (i.e., peer-reviewed publications)	
0-30	47 (64)
31-100	22 (30)
101-400	5 (6)

PDs had, on average, been in their role for 6.7 years (95% confidence interval [CI] 5.3-9.2; range 1-26 years). In response to the question about involvement in any other research collaboratives, 27% (20/74) reported one or more groups, while 73% (54/74) reported no involvement. There were several research collaboratives across orthopedic subspecialties identified including: Tumor (MORI, Musculoskeletal Oncology Research Initiative), Trauma (METRC, Major Extremity Trauma Research Consortium), and Pediatrics (IPSG, International Perthes Study Group; PSSG, Pediatric Spine Study Group).

Benefit, ability, and support of an orthopedic education research collaborative

Ninety-nine percent (73/74) of orthopedic faculty leadership stated that resident education and faculty development research projects would benefit from an orthopedic education research collaborative. Eighty-one percent (60/74) of faculty agreed that a formal collaborative would substantially benefit the ability to do research. Orthopedic residency leaders (91%, 67/74) would support the creation of an orthopedic collaborative.

Eighty-four percent (62/74) of the total responses were from PDs. Among PDs, 92% (57/62) would support the creation of an orthopedic education research collaborative. In comparison to unsupportive (*without support*) respondents, younger age (*P *= 0.006), 15 or fewer years in practice (*P *= 0.04), and having 0 to 100 peer-reviewed publications (*P *= 0.047) were associated with support for an orthopedic collaborative (Table 2). Participants aged 55 years or older had the highest proportion (71%) of the *without support* group compared to those aged 35 to 44 (14%) and 45 to 54 (14%) years (*P *= 0.006). Similarly, participants who had been in practice for a longer period (>15 years) comprised a higher proportion of the *without support* group compared to those who had been in practice for ≤15 years (85.7 versus 14.3%; *P *= 0.03). Among the *without support* group, clinical faculty comprised the highest proportion of respondents unsupportive of an orthopedic collaborative (50%) compared to Assistant (0%), Associate (11%), and full Professors (6%) (*P *= 0.02).

**Table 2:** Factors grouped by support of an orthopedic collaborative. *Statistical significance at *P *< 0.05. All percentages are calculated by row.

Factor	Yes, *n* (%)	No, *n* (%)	P
Total	67 (91)	7 (9)	
Age (years)			0.006*
35-44	30 (97)	1 (3)	
45-54	25 (96)	1 (4)	
55+	12 (71)	5 (29)	
Gender			0.07
Women	15 (100)	0 (0)	
Men	52 (88)	7 (12)	
Region			0.54
Northeast	15 (88)	2 (12)	
South	24 (96)	1 (4)	
Midwest	15 (83)	3 (17)	
West	13 (93)	1 (7)	
Program size (average number of residents per year)			0.07
2-3	11 (79)	3 (21)	
4-6	48 (96)	2 (4)	
7 or more	8 (80)	2 (20)	
Years in practice			0.04*
>15 years	29 (83)	6 (17)	
≤15 years	37 (97)	1 (3)	
Academic rank			0.02*
Clinical Faculty	2 (50)	2 (50)	
Assistant Professor	16 (100)	0 (0)	
Associate Professor	33 (89)	4 (11)	
Professor	16 (94)	1 (6)	
Number of peer-reviewed publications			0.047*
0-30	43 (91.5)	4 (8.5)	
31-100	21 (95)	1 (5)	
101-400	3 (60)	2 (40)	

Concerns and challenges

**Figure 1:**
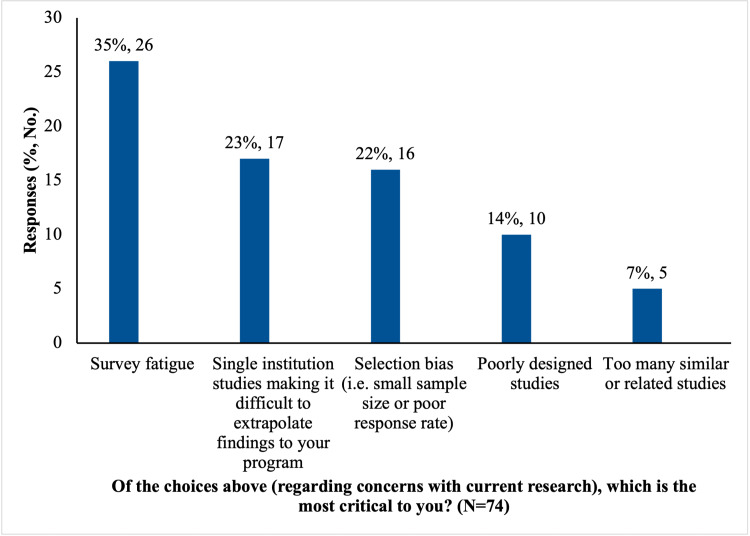
Critical concerns with current orthopedic research climate.

The most frequently reported concern with the current state of orthopedic education research was *survey fatigue* (76%, 56/74). Survey fatigue was also selected as the most critical concern regarding current research (Figure 1). The majority of respondents reported concerns regarding selection bias (68%, 50/74) and the inability to extrapolate findings to other programs based on single-institution data (65%, 48/74; Figure 1).

Challenges faced by respondents in attempting to perform resident education research were comparable across the group regardless of the support level for a collaborative group. A majority of faculty cited *insufficient time* as a challenge to performing resident education or faculty development research (78%, 58/74). Other reported challenges include inexperience in this type of research (41%, 30/74), few grant opportunities (38%, 28/74), and no industry funding (36%, 27/74; Table 3). There were no significant differences in responses to challenges based on gender, age, academic, rank, or program size.

**Table 3:** Challenges in conducting orthopedic resident education or orthopedic faculty development research. Note: Each percentage represents the number of people out of the total group who found that item to be a challenge. AAOS, American Academy of Orthopedic Surgeons

	Selected (%)
Insufficient time	78
Inexperience in this type of research	41
Few grant opportunities	38
No industry funding	36
Lack of like-minded colleagues interested in these topics	31
No recognized submission category for abstract to annual AAOS meeting	27

Recommendations for an orthopedic research collaborative

**Figure 2:**
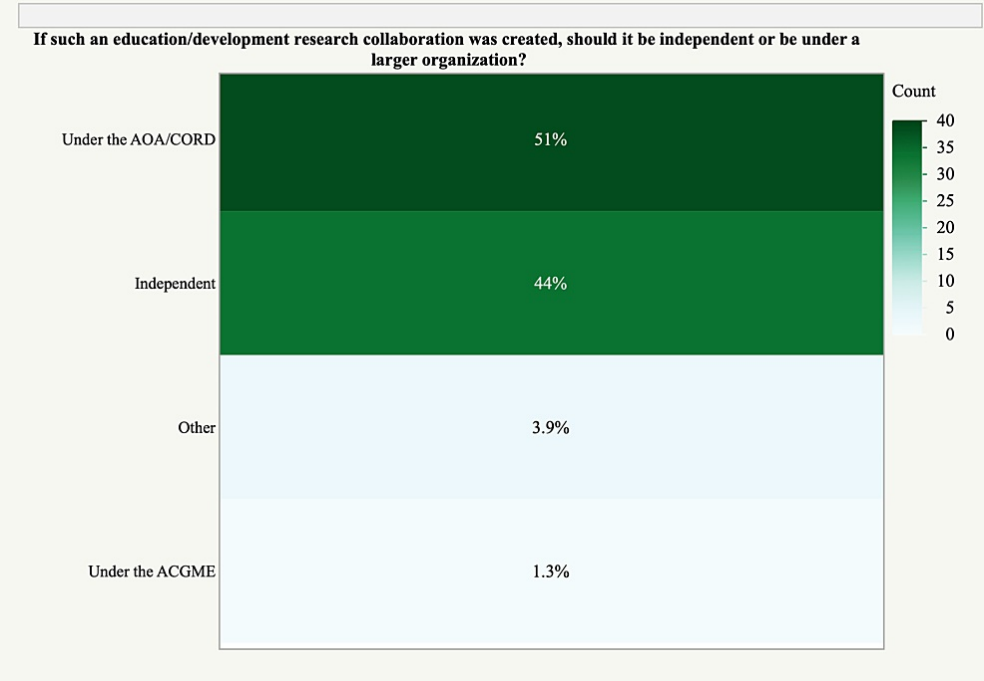
Proposed governance of an orthopedic research collaborative. AOA/CORD, American Orthopedic Association/Council of Orthopedic Residency Directors; ACGME, Accreditation Council for Graduate Medical Education

Multiple recommendations were identified regarding the creation of a potential orthopedic education research collaborative. The benchmark number of survey-based projects for residents to complete was a median of 2 (mean ± SD, 1.74 ± 0.95) per month and for faculty was also a median of 2 (mean ± SD, 1.68 ± 0.97) per month. The majority of respondents (93%, 69/74) believed that incentivization with shared authorship was appropriate if the International Committee of Medical Journal Editors (ICMJE) authorship criteria were met. Regarding structural oversight or governance, 50% (37/74) supported oversight by AOA/CORDs, while 45% (33/74) preferred to operate independently (Figure 2).

## Discussion

This study demonstrated that orthopedic residency leaders’ attitudes toward a collaborative education research group are supportive. The inception of a multicenter orthopedic research collaborative is consistent with responses underscoring the notion that there is a likely benefit associated with a group of like-minded individuals interested in resident education and faculty development. Factors specifically associated with support for an orthopedic collaborative included younger age, less than 15 years in practice, and higher academic rank. Except for a higher academic rank, these factors are somewhat surprising - the authors specifically hypothesized that greater academic/research experience would be associated with support. Additional findings highlighting greater support among younger orthopedic faculty and among surgeons with fewer years in practice suggest that support for a research collaborative may be rooted in increased favorability of academic collaboration, encouraged early and often throughout orthopedic training and into practice.

While high-quality, generalizable findings can shape national policies using real-world evidence from individuals who influence training, many studies are limited by results generated by single-institution input. However, collaborative research groups provide organization and improve the production of high-quality research findings that can be used in a broad setting [14,28]. In other fields, collaborative education research groups generated broadly applicable research [16]. The Medical Education Research Group, a community specifically interested in Emergency Medicine residency education and faculty development, describes a structured approach to increase the quality and number of publications that can broadly be implemented [12]. Tangible results from the Medical Education Research Group over four years include 46 publications, 13 MedEdPORTAL publications, and five faculty promotions [12]. Success is credited to valuable mentorship, effective teamwork, and the collaborative identification of education gaps in program curricula across the country [12]. The authors hypothesize that the coordination of a multi-institution collaborative in orthopedics can effectively use these principles to limit the redundancy of study topics and maximize the validity of studies, thereby mitigating some of the issues related to study fatigue and inadequate time.

In this study, a majority of faculty cited insufficient time as the greatest challenge to performing resident education and faculty development research. While single-institution, survey-based studies can provide notable findings that impact individual opinion or public policy, these studies are often limited by issues related to generalizability and sampling issues [29]. Within the context of literature, the findings of this study suggest that there is a need for an innovative approach to collaborate among leadership at multiple institutions to minimize survey-based study fatigue, elevate study quality, and provide results that can be implemented in a geographically broad group of programs. The development and distribution of national survey-based studies to examine diversity, education, health, and leadership interventions is likely to be widely beneficial. On average, respondents suggested that national survey-based studies with broader implications for orthopedic education endorsed by the collaborative be limited to two requests for residents and two for faculty per month. Similar studies can be developed that are distributed to both residents and faculty. Following critical review, investigators may have the opportunity to improve the proposal with feedback from the group.

Respondents provided additional suggestions for the successful creation and operation of a research collaborative in orthopedics regarding authorship and governance. A majority of respondents indicated that incentivization with shared authorship would be one way to generate involvement in research collaboration. Indeed, the Association for Surgical Education (ASE) and its annual conference illustrate the practicality and benefit of such collaboratives rooted in surgical education in the United States. The utility of skills-based workshops in conjunction with plenary academic sessions not only facilitates collaboration but also fosters networking opportunities and the development of hands-on surgical skills [30]. We also look to the SIMPL collaborative as a *gold standard* model for growth - although quality improvement remains at the heart of SIMPL, collaboration utilizing data from its mobile resident feedback platform has resulted in over 40 publications across 13 surgical specialties [22]. It is, however, worth noting that the cost, and financial burden of membership, may be prohibitive in creating and maintaining quality improvement collaboratives. In contrast, the authors intend to utilize the results of our survey towards the inception of a cost-free surgical research initiative. As such, we strive to bridge the gap between academic and community-based orthopedic surgery programs, setting the stage for equitable collaboration.

Furthermore, respondents were largely split regarding governance between AOA/CORD oversight and independent operation. While working within a larger organization may provide opportunities for the group to advance timely topics, the ability for independent operation may promote freedom to pursue questions of interest to a broader community.

Limitations

There are several limitations inherent to this study. Our survey was delivered to orthopedic surgery residency PDs electronically via contact information provided by the ACGME. Although 62 PDs responded to our survey, we are unable to verify whether program-specific contact information provided by the ACGME is current or whether our survey was distributed to PDs themselves versus program support staff; therefore, we are unable to determine the overall response rate of our survey. This study also includes potential response bias. Differences between the respondents who completed the survey and the target population are not limited to sociodemographic factors but can include attitudes toward residency education research and the institutions involved. Response bias may also relate to volunteers being surveyed, as volunteers might be less likely to respond with opinions perceived as less acceptable. As a study of a single point in time, the authors do not make any claims about causality or inferences about the proposed collaborative group's precise ability to meet the needs of the faculty from across the country with potentially more granular preferences that were not surveyed. In light of the inherent biases in survey studies, best practices for survey research to deal with the critical problems that can arise are necessary [29]. The underlying purpose of a collaborative group in the orthopedic training community would be to improve the quality of resident/faculty education and professional development-related research to promote better outcomes for patients. As such, our study sought to elucidate the benefit of an orthopedic research collaborative rooted in survey-based research studies with the potential for broad impact about orthopedic surgical training in the United States. We recognize that survey-based research only represents a fraction of potential research studies, and additional development of our proposed orthopedic research collaborative will be required to facilitate the completion of ancillary research studies on a national scale.

## Conclusions

Resident education and faculty development research projects will likely benefit from an orthopedic education research collaborative to overcome concerns related to survey fatigue, selection bias, single-institution studies, and challenges, including insufficient time, inexperience in this type of research, and lack of funding. Overall, orthopedic residency leaders support the creation of an education research collaborative. Following a critical review of study proposals, an organized group of leaders in orthopedic surgery can selectively distribute up to two survey-based studies per month to residents and faculty. This could progress to more advanced experimental studies, including prospective education studies. Concurrently, the proposed orthopedic collaborative represents a promising path to develop robust, evidence-based research in orthopedic education.

## Figures and Tables

**Figure 3:**
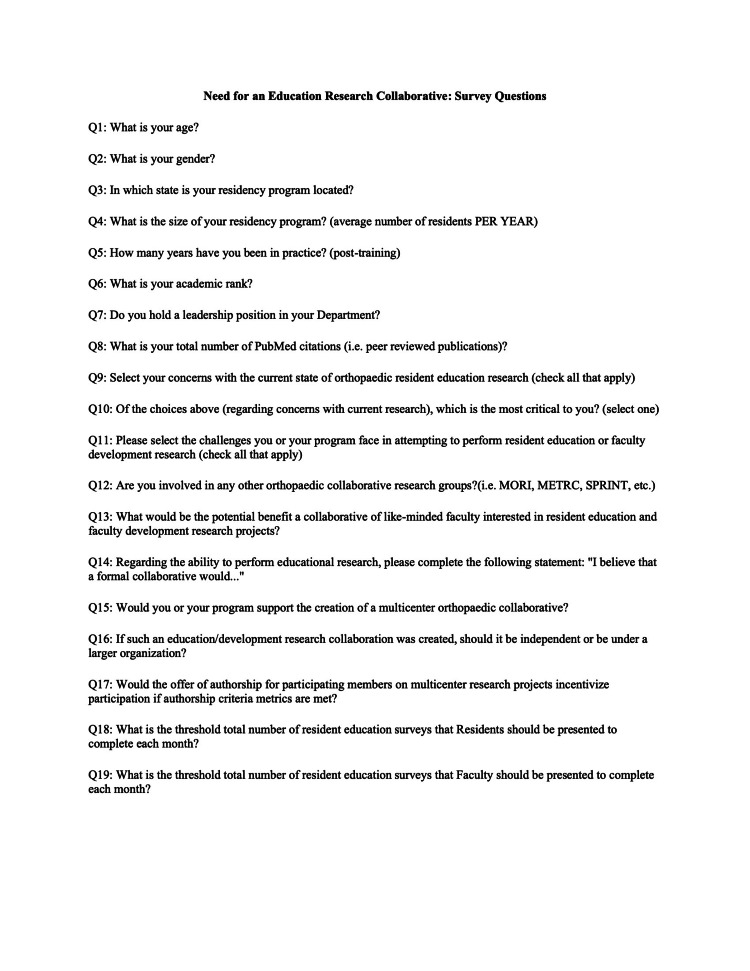
Need for COERG: survey questions. COERG, Collaborative Orthopedic Educational Research Group
